# An ancestral allele of grapevine transcription factor *MYB14* promotes plant defence

**DOI:** 10.1093/jxb/erv569

**Published:** 2016-02-02

**Authors:** Dong Duan, Sabine Fischer, Patrick Merz, Jochen Bogs, Michael Riemann, Peter Nick

**Affiliations:** ^1^Molecular Cell Biology, Botanical Institute 1, Karlsruhe Institute of Technology, Kaiserstr. 2, D-76131 Karlsruhe, Germany; ^2^ College of Life Sciences, Northwest University, Xi’an 710069, China; ^3^Institute of Molecular Genetics, Johannes Gutenberg-University Mainz, J.-J.-Becherweg 32, D-55128 Mainz, Germany; ^4^Dienstleistungszentrum Ländlicher Raum Rheinpfalz, Breitenweg 71, Viticulture and Enology Group, D-67435 Neustadt, Germany; ^5^Fachhochschule Bingen, D-55411 Bingen am Rhein, Germany

**Keywords:** flg22, grapevine (*V. sylvestris*), *MYB14*, *Plasmopara viticola*, stilbene synthase, UV.

## Abstract

Stilbene synthase is a key enzyme for the production of the phytoalexin resveratrol. Some clones of *Vitis sylvestris*, a wild European grapevine species which is almost extinct, have been shown to accumulate more resveratrol in response to different forms of stress. In the current study, we asked whether the induction of stilbene synthase transcripts in Hoe29, one of the *V. sylvestris* clones with elevated stilbene inducibility, might result from the elevated induction of the transcription factor *MYB14*. The *MYB14* promoter of Hoe29 and of Ke83 (a second stilbene-inducible genotype) harboured distinct regions and were applied to a promoter–reporter system. We show that stilbene synthase inducibility correlates with differences in the induction of *MYB14* transcripts for these two genotypes. Both alleles were induced by UV in a promoter–reporter assay, but only the *MYB14* promoter from Hoe29 was induced by flg22, consistent with the stilbene synthase expression of the donor genotypes, where both respond to UV but only Hoe29 is responsive to *Plasmopara viticola* during defence. We mapped upstream signals and found that a RboH-dependent oxidative burst, calcium influx, a MAPK cascade, and jasmonate activated the *MYB14* promoter, whereas salicylic acid was ineffective. Our data suggest that the Hoe29 allele of the *MYB14* promoter has potential as a candidate target for resistance breeding.

## Highlight

The molecular mechanisms underlying the elevated inducibility of stilbene in pathogen-resistant *Vitis sylvestris* can be explained by the increased inducibility of the *MYB14* promoter.

## Introduction

Grapevine is an economically important crop, which is highly susceptible to various biotic diseases, and, therefore, requiring extensive plant protection measures. Viticulture accounts for 70% of the European fungicide consumption. In order to get the ‘pure wine’ with fewer chemicals, more environmentally friendly approaches are warranted. An important element for sustainable viticulture is resistance breeding using resistance factors originating from North American grapes (Gessler *et al.*, 2011).

These resistance factors have to be understood in context with the complex evolution of plant immunity: The plant innate immune system is composed of two distinct layers (Jones and Dangl, 2006). The first layer exploits ubiquitous molecules in pathogenic microorganisms, termed pathogen-associated molecular patterns (PAMPs), to recognize pathogen attack by receptors at the plasma membrane and to activate a basal, so-called PAMP triggered immunity, PTI (Jones and Dangl, 2006; Boller and He, 2009). Most of these PAMPs are essential for the life cycle of the invader, such that the PAMPs are preserved. Pathogens that have undergone coevolution with their hosts, have usually developed an alternative strategy: They can quell PTI by injecting chemical signals, so-called effectors, into the host cell and thus reinstate pathogenicity, which is termed effector-triggered immunity, ETI (Takken and Tameling, 2009). This ETI has evolved during a long battle between the pathogen and the host plant. The molecular mechanisms underlying these two layers of plant immunity differ, but can also overlap (Thomma *et al.*, 2011), which contributes to the complexity of the plant–pathogen interaction.

The economically important grapevine pathogen Downy Mildew (*Plasmopara viticola*) has co-evolved over millions of years together with wild American grapes, such that these wild grapes had enough time to evolve ETI and, therefore, can cope with these pathogens. The recent discovery of *Plasmopara viticola* strains that can infect specific grapevine genotypes (Rouxel *et al.*, 2013; Gómez-Zeledón *et al.*, 2013) support the hypothesis that the resistance of these American grapes is based upon a canonical ETI. However, these wild grapes are not suited for winery, due to their unpleasant ‘foxy taste’ (off-flavour). As a strategy to separate the desired immunity from the undesired flavour, those wild grapes have been crossed with cultivated grape varieties. This strategy has been successful and has culminated in economically important new varieties with good resistance against downy mildew (*P. viticola*). However, the rising success of these new varieties is expected to initiate the next round of the evolutionary warfare. In fact, the resistance conferred by the genetic factor ‘Resistance to *P. viticola* 3 (Rpv3)’ which forms the base of most current disease-resistant grapevine varieties, has already been observed to become eroded by new strains of *Plasmopara* (Peressotti *et al.*, 2010; Gessler *et al.*, 2011; Gómez-Zeledón *et al.*, 2013).

As a strategy to render the success of resistance breeding more sustainable, new sources of resistance are required. In this context, it might also be rewarding to exploit factors stimulating the basal immunity (PTI). The ancestor of cultivated grapevine, *V. vinifera* L. ssp. *vinifera*, the European Wild Grape (*V. vinifera* L. ssp. *sylvestris* Hegi) lacks a history of coevolution with these pathogens and, therefore, should not harbour any ETI-like defence against downy mildew. Nevertheless, many genotypes of the European Wild Grape show good tolerance against several grapevine diseases (Tisch *et al.*, 2014), such as downy mildew (*P. viticola*), powdery mildew (*Erysiphe necator*), and black rot (*Guignardia bidwelli*), which means that some genotypes of *V. sylvestris* might command a more efficient basal immunity. In fact, during previous work, we have identified several genotypes of *V. sylvestris* with a strong induction of antimicrobial stilbenes in response to either a short pulse of UV irradiation or to infection with *P. viticola* (Duan *et al.*, 2015) indicating that early signals shared between these two stress factors must be involved. These genotypes were also endowed with a partial resistance to downy mildew and, therefore, have potential as new breeding resources to be exploited for sustainable viticulture in the future.

Stilbenes, as important phytoalexins, are a central factor for the basal immunity of grapevine. Transcription of the key enzyme stilbene synthase (*StSy*) can be activated by the PAMP flg22 (Chang and Nick, 2012) as part of PTI, but also by the bacterial trigger Harpin (mimicking an ETI-like pattern of defence). Although both immunity responses culminate in an accumulation of stilbene synthase transcripts and share a part of early signalling events, they differ in perception, the role of oxidative burst, and integration into a qualitatively different output of stilbene metabolites. The transcriptional regulation of the stilbene biosynthetic pathway is mediated by two R2R3-MYB-type transcription factors, *MYB14* and *MYB15* that were shown to activate the stilbene synthase promoter (Höll *et al.*, 2013). In our previous work (Duan *et al.*, 2015), the *V. sylvestris* genotypes Hoe29 and Ke83 were found to be endowed with an elevated stilbene inducibility in response to UV light, which was correlated with a strong induction of stilbene synthase transcripts. Interestingly, in response to inoculation with *P. viticola*, stilbene synthase transcripts were elevated for Hoe29, but not for Ke83.

In the current study, we test the idea whether the strong inducibility of stilbene synthase transcripts in Hoe29 might result from the elevated induction of *MYB14* and *MYB15*. Specific differences were discovered by Illumina-based next-generation sequencing and confirmed by cloning for the *MYB14* promoter of Hoe29, whereas *MYB15* did not reveal obvious changes. Both *sylvestris* genotypes shared certain *MYB14* promoter domains (whereas they differ in others), which were absent from the promoter of the cultivated variety Augster Weiss (that is a weak stilbene producer). Using a promoter–reporter assay in grapevine suspension cells (Höll *et al.*, 2013), we show that differences in the inducibility of the *MYB14* promoter can account for the stress-specific differences in the expression of stilbene synthase observed between the three genotypes (Hoe29, Ke83, and Augster Weiss). Although both *sylvestris* genotypes show good UV inducibility of *MYB14*, only the Hoe29 allele of this promoter is competent for induction by PTI (triggered by flg22). We also mapped known signalling events for PTI, such as dependence on NADPH oxidase (RboH) or induction by jasmonic acid, and calcium influx. Our data suggest that a particular region in the promoter of a specific *sylvestris* allele of *MYB14* harbours potential as a candidate target for resistance breeding.

## Materials and methods

### Plant materials

The *Vitis vinifera* ssp. *sylvestris* genotypes ‘Ke83’ and ‘Hoe29’, as well as the *V. vinifera* cultivar Augster Weiss used in this study, have already been described in Ledesma-Krist *et al.* (2014) and Duan *et al.* (2015). All accessions are maintained as living specimens in the grapevine collection of the Botanical Garden of the Karlsruhe Institute of Technology (Olmo, 1976). For DNA and RNA extraction, the leaves were harvested from greenhouse-grown plants cultivated at a day/night temperature of 22/18 °C and a 14/10h light/dark photoperiod.

### Preparation of leaf samples

The fully expanded leaves were excised and subjected to UV-C stress as described in Duan *et al.* (2015). The leaves of the different genotypes were harvested at different time points after the treatment: C (control fresh leaf, without UV-C treatment), 0.5h, and 6h, respectively, immediately frozen in liquid nitrogen, and stored at −80 °C until RNA extraction.

Downy mildew (*P. viticola*) infection was carried out as described previously (Genet *et al.*, 1997; Kortekamp, 2006; Duan *et al.*, 2015). Three experimental situations were used: fresh leaf without inoculation (C), control leaf incubated in the absence of *P. viticola* but kept under the same conditions (120 h-C), and controlled inoculation with a suspension of *P. viticola* and incubation for 120h (120 h-S), respectively. The leaf material was immediately frozen in liquid nitrogen and stored at −80 °C until RNA extraction.

### cDNA synthesis and quantitative Real-Time RT-PCR

Total RNA was isolated using the Spectrum^TM^ Plant Total RNA Kit (Sigma, Deisenhofen) according to the protocol of the manufacturer. The extracted RNA was treated with a DNA-free DNase (Qiagen, Hilden, Germany) to remove any potential contamination by genomic DNA. The mRNA was transcribed into cDNA using the M-MuLV cDNA Synthesis Kit (New England Biolabs; Frankfurt am Main, Germany) according to the instructions of the manufacturer. The RNase inhibitor (New England Biolabs; Frankfurt am Main, Germany) was used to protect the RNA from degradation. The amount of RNA template was 1 μg.

Quantitative real-time RT-PCR was performed on an Opticon 2 system (Bio-Rad, München) as described by Svyatyna *et al.* (2014). To compare the mRNA expression levels between different samples, the *C*_t_ values from each sample were normalized to the value for the *EF-1α* internal standard obtained from the same sample. For each triplicate, these normalized *C*_t_ values were averaged. The difference between the *C*_t_ values of the target gene X and those for the *EF-1α* reference R were calculated as follows: △C_t_(X)=C_t_(X)–C_t_(R). The final result was expressed as 2^–△Ct(X)^. Each experiment was repeated with three biological replicates.

### Illumina next generation sequencing and data analysis

Genomic DNA was extracted from young leaves of Hoe29 using the DNeasy Plant Mini Kit (Qiagen, Hilden, Germany) following the instructions of the manufacturer. For Illumina Next Generation Sequencing, 1.5 μg genomic DNA were sheared using Covaris (Woburn, Massachusetts), and the subsequent library preparation was carried out with the TruSeq DNA LT Sample Prep Kit (Illumina Inc., San Diego, CA). The resulting library was sequenced in a 100bp paired-end run on an Illumina HiSeq2000 generating approximately 200 million reads (IMSB, Mainz, Germany). The subsequent analysis of NGS data was conducted with the CLC bio Genomics Workbench (Aarhus, Denmark). Raw reads were filtered for quality (*P*=0.01, no ambiguity nucleotides) and adapter trimmed. *De novo* assembly of remaining trimmed reads (approximately 187 million reads) was conducted using standard parameters. The resulting contigs served as the database for BLASTn searches using the *MYB14* sequence of PN40024 (NW_003724037.1) as a query. Trimmed reads were mapped to cloned promoter sequences of Hoe29, Ke83, and Augster Weiss with varying lengths and similarity fractions: 1.0/1.0; 0.98/0.98; 0.96/0.96.

### Cloning of the *MYB14* promoter

In order to conduct the transient expression assay, the *MYB14* fragments of genomic DNA – Hoe29, Ke83, and Augster Weiss were amplified with the Phusion DNA polymerase (NEB, Frankfort, Germany), including the promoter sequences of Hoe29 (1351bp), Ke83 (1245bp), and Augster Weiss (1347bp). The primers *MYB14*-F (5′-CTACTGACGTGCACTAGCCT-3′) and *MYB14*-R (5′-GCAG AGTGAAAGTGCAACACG-3′) were designed according to the reference sequence in GenBank (NW_003724037.1). The isolated sequences were compared with the database sequence using the multiple sequence alignment program ClustalW2 (http://www.ebi.ac.uk/Tools/msa/clustalw2/). After the alignments, specific primers for GATEWAY cloning (see Supplementary Table S1 at *JXB* online) were designed to amplify the chosen promoter sequence of Hoe29, Ke83, and Augster Weiss using the GATEWAY^®^-Cloning technology (Invitrogen Corporation, Paisley, UK). The promoter regions of these three genotypes were ligated into the luciferase vector pLuc (Horstmann *et al.*, 2004) and verified by DNA sequencing (GATC Biotech, Cologne, Germany).

### Transient transfection and dual-luciferase assay

A transient assay using a cell suspension culture of a ‘Chardonnay’ petiole callus maintained on Grape Cormier (GC) medium (Bao Do and Cormier 1991), was performed as described previously (Czemmel *et al.*, 2009; Merz *et al.*, 2014) with minor modifications as follows: after a transfection (48h), the cells were harvested after a treatment with different stresses at specified time points, and then lysed by grinding on ice in 200 μl of 2× passive lysis buffer (PLB, Promega, Madison, Wl) for 1.5min using a pestle and mortar. After centrifugation of the lysates for 1min at 1 000 *g*, luciferase activities were measured with the dual-luciferase reporter assay system (PJK, Kleinblittersdorf, Germany), by the sequential addition of 50 μl Beetle Juice and Renilla Glow Juice to 20 μl of the individual lysate supernatants. Light emission was measured with a lumat LB9507 Luminometer (Berthold Technologies, Bad Wildbad, Germany). The specific promoter linked to a firefly luciferase gene was co-bombarded with the Renilla luciferase plasmid pRluc as an internal standard and the relative luciferase activity was calculated as the ratio between the firefly and Renilla (control) luciferase activity. All transfection experiments were performed in triplicate with similar relative ratios to the respective control.

### Treatment of the cells for transient promoter assays

For the UV-C experiment, the cells were treated for 2min at a distance of 12.5cm by UV-C (15W, OSRAMHNS, OFR) and then harvested at 3h (Duan *et al.*, 2015). The bacterial peptide flg22 QRLSTGSRINSAKDDAAGLQIA (Felix *et al.*, 1999), a 22-amino-acid peptide, was purchased from a commercial producer (Antikörper online, Aachen, Germany) and diluted in sterile H_2_O as a stock solution of 1mM. Diphenyleneiodonium chloride (DPI) (Sigma-Aldrich, Germany) was dissolved in dimethyl sulphoxide (DMSO) as a stock solution of 10mM. The calcium ionophore A23187 (Sigma-Aldrich, Germany) was diluted in DMSO as a stock solution of 50mM. Gadolinium chloride (GdCl_3_) (Sigma-Aldrich, Germany) was used as an inhibitor of mechanosensitive calcium channels and diluted with DMSO to a 20mM stock solution. PD98059, a chemical inhibitor for the mitogen-activated protein kinase (MAPK) cascade (Sigma-Aldrich, Deisenhofen, Germany), was dissolved and sterilized into a 100mM stock solution in DMSO. Salicylic acid (SA) (Sigma-Aldrich, Germany) and (±)-jasmonic acid (JA) (Sigma-Aldrich, Germany) were dissolved in ethanol (EtOH) to obtain stock solutions of 50mM and 500mM, respectively. The inhibitor 1-phenylpyrazolidinone (phenidone) (Sigma-Aldrich, Germany) was dissolved in DMSO to obtain a 2M stock solution. Polyoxyethylen-20-sorbitan monolaurate (Tween^®^ 20), required in a low concentration (1 ‰) to keep phenidone soluble, was obtained from Carl Roth in Germany. All treatments were accompanied by appropriate solvent controls, and the maximal concentration of solvent used in the test samples did not exceed 1‰.

## Results

### Specific alleles of the *MYB14* promoters in *sylvestris*

During the comparison of the *sylvestris* genotype Hoe29, which has high stilbene-inducibility (Duan *et al.*, 2015), significant differences in the region of the *MYB14* promoter were discovered with respect to the reference genome (established for the *vinifera* variety ‘Pinot Noir’) by next-generation sequencing. This region was, therefore, cloned from the two *sylvestris* genotypes Hoe29, and Ke83 (a second *sylvestris* genotype with high stilbene-inducibility), together with the ancient *vinifera* variety ‘Augster Weiss’, which is male-sterile and, therefore, often used for breeding. The alignment of these promoters showed significant differences that were then analysed with respect to predicted promoter motives using the PlantCARE algorithm (Lescot *et al.*, 2002).

**Fig. 1.**
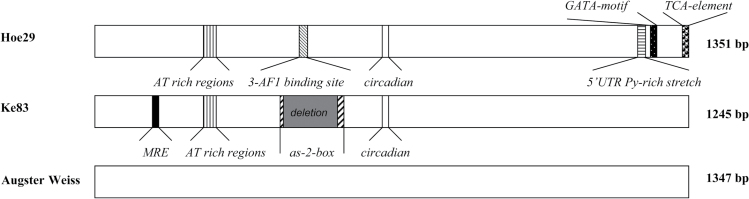
Comparison of *MYB14* promoters in the two *sylvestris* genotypes Hoe29 and Ke83, along with the cultivated variety Augster Weiss. 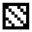 3-AF1 binding site (11bp): a light-responsive element. 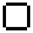 circadian (10bp): *cis*-acting regulatory element involved in circadian control. 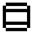 5′UTR Py-rich stretch (10bp): *cis*-acting element conferring high transcriptional levels. 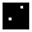 GATA-motif (7bp): part of a light-responsive element. 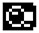 TCA-element (10bp): *cis*-acting element involved in salicyclic acid responsiveness. 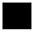 MRE (7bp): *MYB* binding site involved in light responsiveness.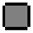 Deletion (107bp) in Ke83 compared with Hoe29 and Augster Weiss. 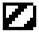 as-2-box (10bp): involved in shoot-specific expression and light responsiveness. 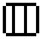 AT rich regions (19bp) in Hoe29 and Ke83.

In Fig. 1, the significant differential *cis*-elements are summarized for the *MYB14* promoters of Hoe29 and Ke83, compared with Augster Weiss (the full alignment with the details on these *cis*-elements are given in Supplementary Fig. S1 at *JXB* online). The Hoe29 promoter harboured a specific 5′-UTR Py-rich stretch, which was absent in the alleles from Ke83 and Augster Weiss due to a deletion of 10bp. This *cis*-acting element has been reported to confer high transcriptional levels (Daraselia *et al.*, 1996; Wang *et al.*, 2013). Furthermore, a 19-bp-long AT-rich insertion was found in Hoe29 and Ke83 (ATTTATTAAATTTATTTTT) which has been found to act as an enhancer (Bustos *et al.*, 1989; Sandhu *et al.*, 1998). In addition, several *cis*-elements linked to light responsiveness have also been found, such as a 3-AF1 binding site and a GATA-motif specific for Hoe29; and a MRE and an as-2-box in Ke83. Most notably, there was a big deletion of 107bp in Ke83 removing two putative CAAT boxes present in Hoe29. These putative enhancer elements were also absent in Augster Weiss (although this genotype did not carry the 107bp deletion). In addition, the promoter in Hoe29 displayed a TCA element shown to confer salicylic-acid responsiveness (Goldsborough *et al.*, 1993).

### Responses of *MYB14* to UV-C and *P. viticola*

To verify whether the transcription factor *MYB14* is indu ced under conditions where the stilbene branch of the phenylpropanoid pathway is activated, we followed the transcript levels of *MYB14* in Hoe29, Ke83, and Augster Weiss in response to UV-C and *P. viticola* by quantitative real-time PCR.

**Fig. 2.**
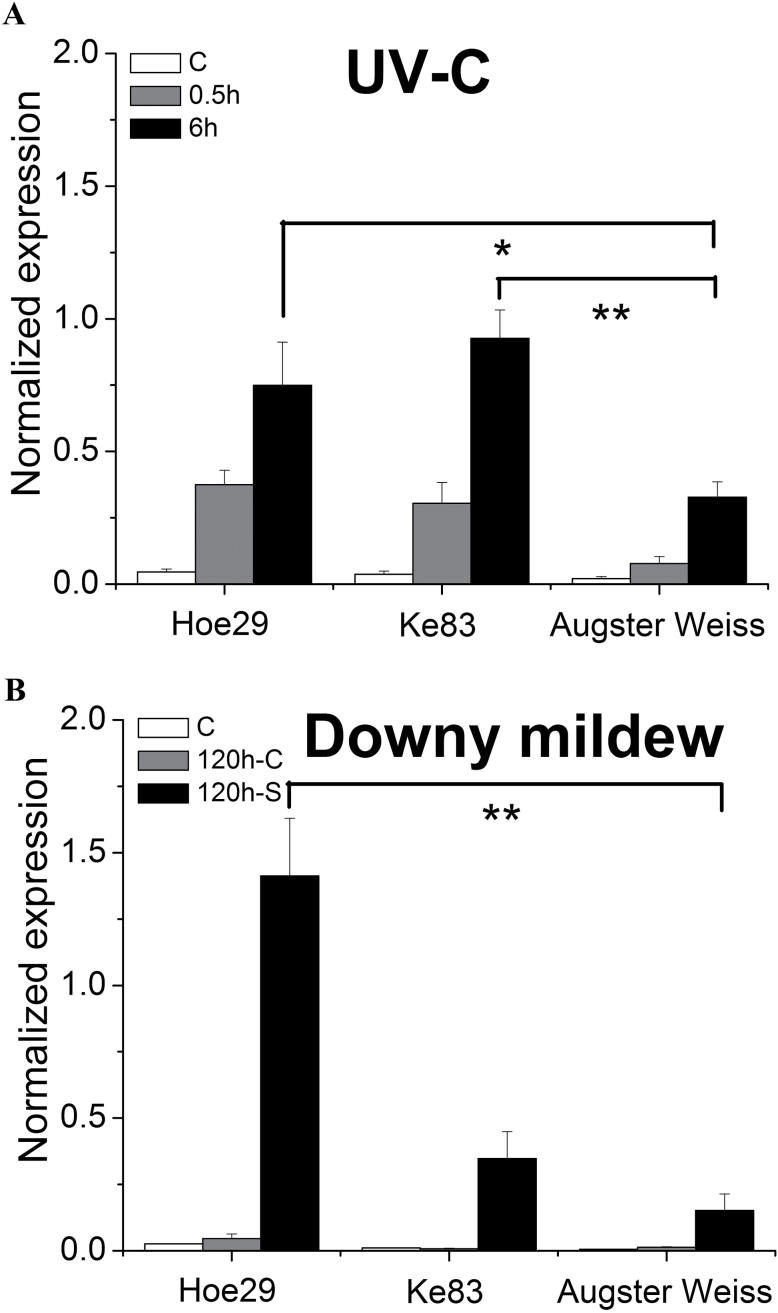
Expression of *MYB14* in response to UV-C and downy mildew. (A) UV-C irradiation for 10min. (B) Downy mildew infection for 120h. Quantification of transcripts by quantitative real-time PCR normalized to the expression of elongation factor *EF1-α*. * indicate differences that are statistically significant on the *P* <0.05 level and ** indicate *P* <0.01 level. Data represent mean values from three independent experimental series, error bars represent standard errors.

As shown in Fig. 2A, for all the genotypes tested, no significant transcript levels can be detected in the controls. However, as early as 0.5h after a UV pulse, these transcripts had been clearly induced, with the response of Hoe29 and Ke83 being much stronger than that of Augster Weiss. This difference was magnified at 6h, when the induction in Hoe29 was 16-fold compared with the control and more than two times compared with Augster Weiss; also in Ke83, this induction was nearly three times higher compared with Augster Weiss.

For infection with downy mildew, the expression of *MYB14* in Hoe29 was strongly induced by 30-fold compared with the control (Fig. 2B), which was more than nine times higher compared with Augster Weiss. The induction in Ke83, although also higher than in Augster Weiss, was not comparable with that in Hoe29.

### Differential activation of *MYB14* promoters from different genotypes

Using a promoter–reporter assay in grapevine suspension cells, we mapped known early signalling events such as the dependence on NADPH oxidase (RboH), or induction by jasmonates and calcium influx to investigate whether the differences in the inducibility of the *MYB14* promoter can account for the stress-specific differences in the expression of stilbene synthase observed between the three genotypes (Hoe29, Ke83, and Augster Weiss).

#### Activation of MYB14 by UV-C requires an oxidative burst

The rapid generation of reactive oxygen species (ROS), termed an oxidative burst, is an early inducible plant response during pathogen invasion or after treatment with elicitors (Wojtaszek, 1997). To test whether the induction of *MYB14* by UV-C requires an oxidative burst, the NADPH oxidase inhibitor DPI was used to quell the increase in ROS abundance following challenge with UV irradiation.

**Fig. 3.**
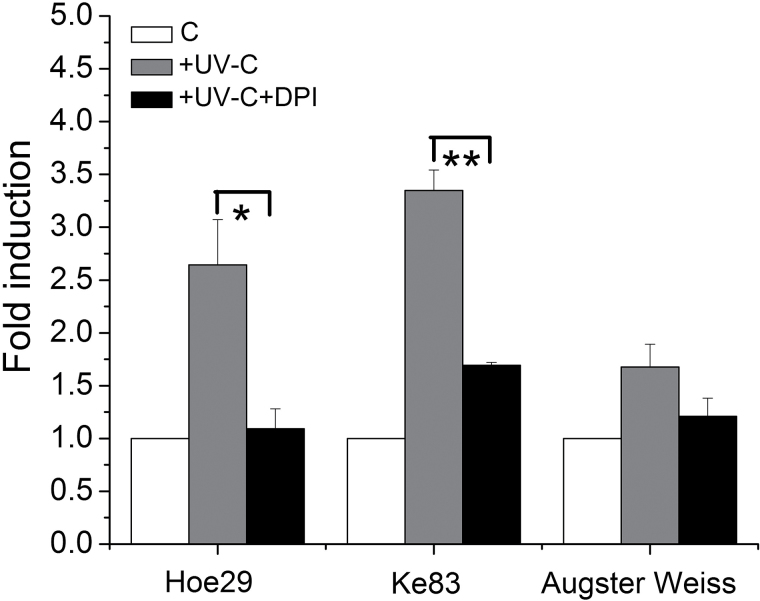
Effect of DPI on promoter activity of *MYB14* in response to UV-C. Values give fold induction levels of promoter activity in the presence of UV-C and UV-C with NADPH oxidase inhibitor (DPI) relative to the respective control (C) (promoter activity without any treatments). +UV-C: fold induction of promoter activity for 3h after the UV-C irradiation for 2min; +UV-C+DPI: fold induction of promoter activity for 3h after the addition of DPI (10 μM) and UV-C for 2min. Values were calibrated against the co-bombarded *Renilla* luciferase plasmid pRluc as an internal standard. * indicate differences that are statistically significant on the *P* <0.05 level and ** indicate *P* <0.01 level. Mean values and standard errors from three independent experimental series.

As shown in Fig. 3, the promoters were activated in all genotypes after UV irradiation, but were stronger in Hoe29 and Ke83 (around 3-fold) compared with Augster Weiss (only around 1.5-fold). Pretreatment with DPI nearly abolished the induction by UV; in Hoe29, the induction was even completely eliminated.

#### Impact of JA and SA on the activation of MYB14 promoters

**Fig. 4.**
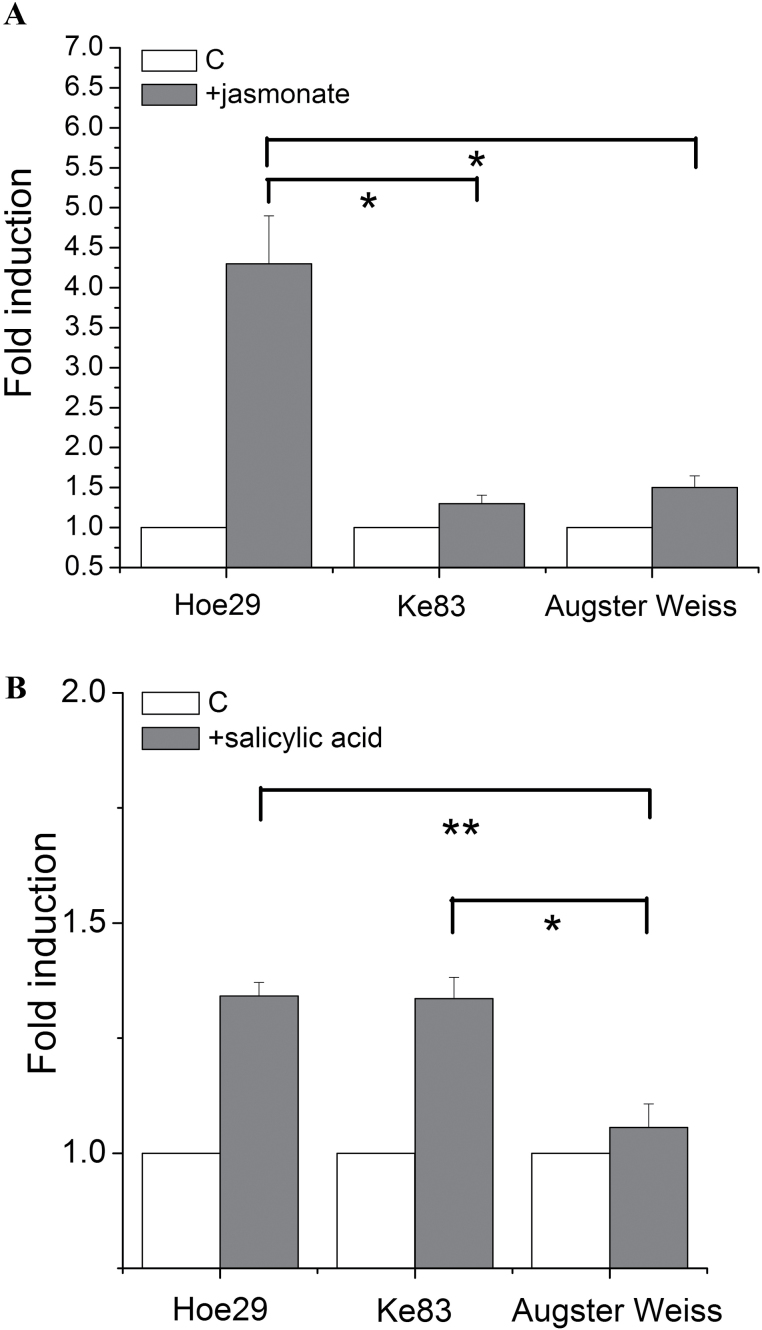
Activities of *MYB14* promoters in response to (±)-jasmonic acid (JA) and salicylic acid (SA). Values show promoter activities relative to the untreated control after the treatments of 50 μM (±)-jasmonic acid (JA) for 4h (A) and 50 μM salicylic acid (SA) for 4h (B), respectively. * indicate differences that are statistically significant on the *P* <0.05 level and ** indicate *P* <0.01 level. Mean values and standard errors from three independent experimental series.

Since jasmonic acid (JA) signalling is activated in response to herbivores, necrotrophic pathogens, and to wounding, we tested whether JA signalling was involved in the activation of the *MYB14* promoters at the early signalling stage, by applying exogenous jasmonates. As shown in Fig. 4A, this induced the promoter activity by around 4-fold in Hoe29, whereas there was hardly any induction in Ke83 and Augster Weiss.

The salicylic acid (SA) pathway acts antagonistically to JA signalling, and is triggered during both PTI and ETI. Since a putative SA-response element had been found in the promoter from Hoe29 (Fig. 1), we therefore tested the effect of exogenous SA. As shown in Fig. 4B, the induction in Hoe29 and Ke83, although significantly higher than in Augster Weiss, was very weak (around 30%) and thus not comparable with the JA induction in Hoe29 (Fig. 4A). This means that while jasmonate can effectively activate the Hoe29 allele of the *MYB14* promoter, SA seems to be fairly marginal.

#### The MYB14 promoters are activated in response to a calcium ionophore

**Fig. 5.**
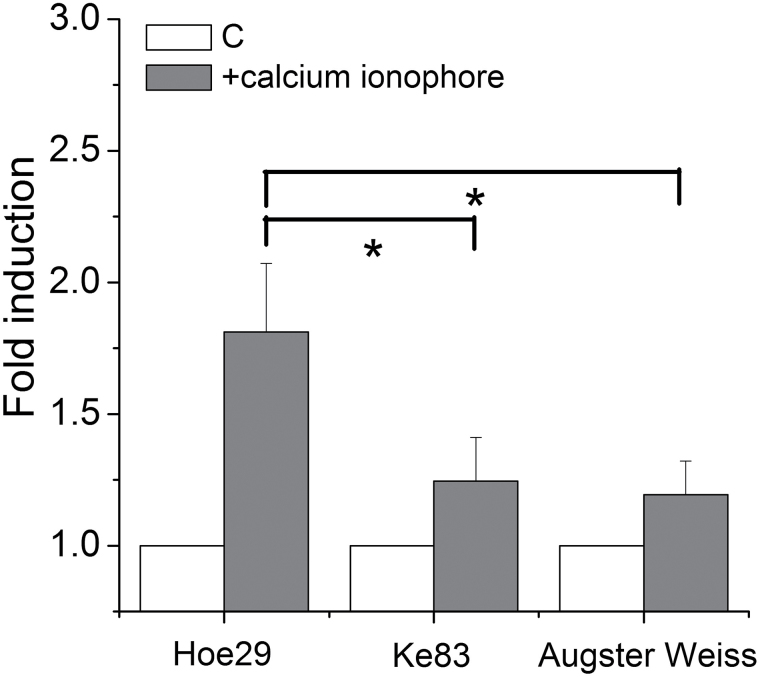
Activities of *MYB14* promoters in response to the calcium ionophore A23187. Values show promoter activities relative to the untreated control after treatment with 50 μM of A23187 for 4h. * indicate differences that are statistically significant on the *P* <0.05 level. Mean values and standard errors from three independent experimental series.

The influx of Ca^2+^ is considered to be the earliest signalling event in basal immunity and we therefore used the antibiotic ionophore A23187 as a probe which can permeate through the cell membrane and release Ca^2+^ in the cytoplasm. This allows calcium to be triggered in the absence of an external stimulus which provides an important tool for functional analysis. To test whether the influx of Ca^2+^ is sufficient for the activation of the *MYB14* promoters, the calcium ionophore was applied. As shown in Fig. 5, this treatment induced promoter activity by around 80% for Hoe29, whereas the induction (although significant) was much weaker (around 20%) in Ke83 and Augster Weiss.

#### The PAMP flg22 can induce the MYB14 promoter in Hoe29 but not in Ke83

**Fig. 6.**
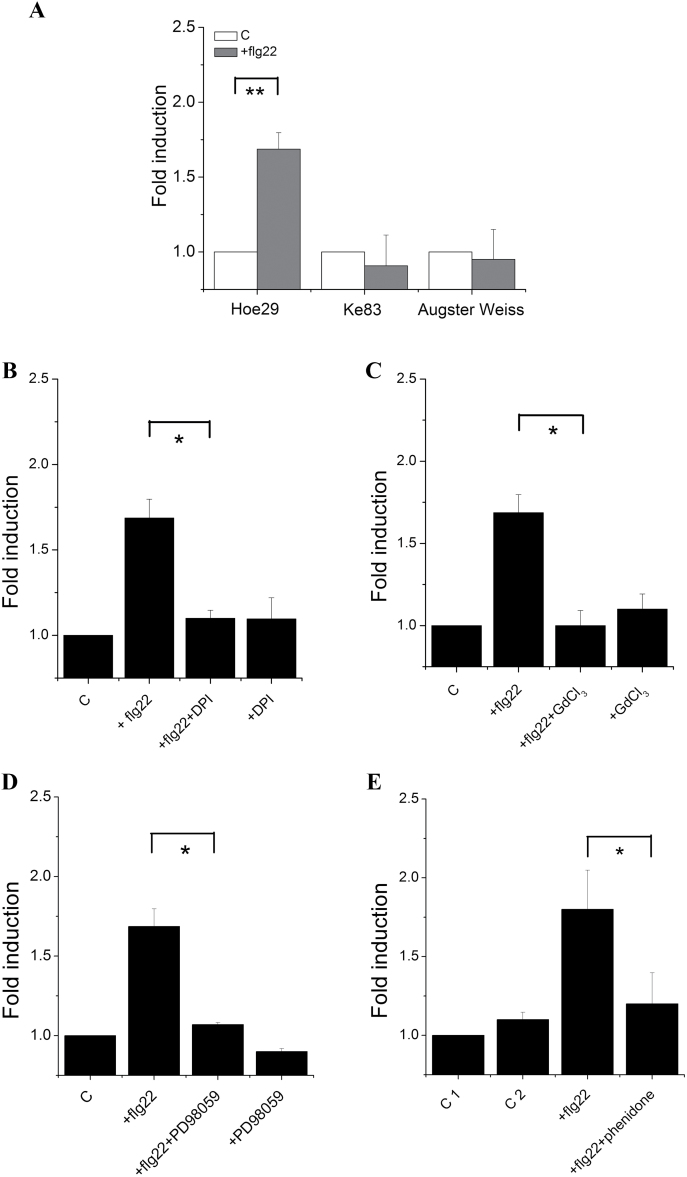
Activities of the *MYB14* promoter from Hoe29 in response to flg22 and the effect of inhibitors on this activity. Values show promoter activities relative to the untreated control after treatment with 1 μM flg22 for 4h (A), the modulation of this activity by pretreatment with: 10 μM of the NADPH oxidase inhibitor DPI (B), 20 μM of the calcium-channel blocker GdCl_3_ (C), 100 μM of the MAPK cascade inhibitor PD98059 (D), and 2mM of the lipoxygenase inhibitor phenidone (E). Activities were measured 4h after the addition of flg22; pretreatments were for30min. In the phenidone experiment (E), 2mM phenidone containing 0.1% Tween^®^ 20 was used, control 1 was pretreated with 0.1% Tween^®^ 20 for 30min alone and then kept for 4h without flg22, control 2 was pretreated with 0.1% Tween^®^ 20 for 30min and then incubated with 1 μM flg22 for 4h. * indicate differences that are statistically significant on the *P* <0.05 level. ** indicate differences that are statistically significant on the *P* <0.01 level. Mean values and standard errors from three independent experimental series.

In order to test whether the stronger induction of *MYB14* by the calcium ionophore in Hoe29 was linked with a higher sensitivity of this allele to basal immunity, the responses to the PAMP flg22 were monitored (Fig. 6A). Treatment with this PAMP induced the *MYB14* promoter activity in Hoe29 by a similar factor (+70%) as treatment with the calcium ionophore. Again, Ke83 and Augster Weiss did not show this induction. Since this pattern suggested that the Hoe29 allele of the *MYB14* promoter was activated by PTI, whereas the Ke83 allele was not, we decided to map known upstream events involved in flg22-triggered basal immunity for Hoe29.

RboH-dependent oxidative bursts are necessary. In order to test whether a RboH-dependent oxidative burst was involved in the activation of the Hoe29 allele of the *MYB14* promoter, DPI was used which almost eliminated the activation in response to flg22 (Fig. 6B). A negative control showed that application of DPI alone did not cause a significant change of activity.

Calcium influx is necessary. Since the activity of a calcium influx channel is essential for the activation of early defence, inhibition of this influx by GdCl_3_, an inhibitor of mechanosensitive calcium channels should therefore suppress the activation of PTI by flg22 (Chang and Nick, 2012). Since a calcium ionophore was able specifically to activate the Hoe29 allele of the *MYB14* promoter of Hoe29 (Fig. 5), we tested whether the specific activation of the Hoe29 allele in response to flg22 required a calcium influx. As shown in Fig. 6C, this was the case. This finding suggests the Ca^2+^ influx is essential for the flg22-induced activation of the *MYB14* promoter.

MAPK signalling is necessary. A mitogen-activated protein kinase (MAPK) cascade is implied in the activation of defence gene expression which can be inhibited in grapevine cell cultures by the specific inhibitor PD98059 (Chang and Nick, 2012). As shown in Fig. 6D, this inhibitor can efficiently suppress the flg22-induced activation of the *MYB14* promoter, demonstrating that MAPK signalling is necessary for this activation.

JA synthesis is necessary. Since exogenous JA could efficiently activate the Hoe29 allele of the *MYB14* promoter (Fig. 4A), we tested whether endogenous jasmonate was necessary for the activation of *MYB14* promoter in response to flg22. Since jasmonate synthesis initiates from a peroxidation of lipids, phenidone, an inhibitor of lipoxygenases, can block the synthesis of JA. As shown in Fig. 6E, a pretreatment with phenidone can efficiently inhibit the flg22-triggered induction of the Hoe29 allele of the *MYB14* promoter. Thus, JA synthesis is necessary for activation.

## Discussion

**Fig. 7.**
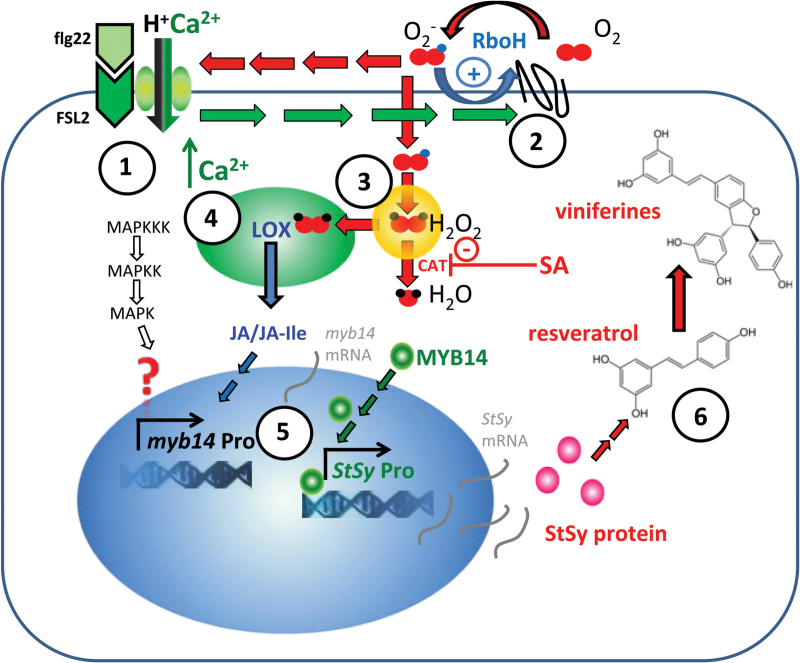
The signature model of immunity signalling. Details are explained in the discussion. 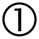 Binding of flg22 to the receptor will therefore result in a substantial increase of cytosolic calcium within a few minutes. 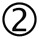 The stimulation of the membrane-bound NADPH oxidase RboH through specific calcium-dependent protein kinases, leading to an apoplastic oxidative burst generating superoxide anions. 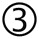 Hydrogen peroxide is generated in the peroxisomes from superoxide. 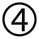 Calcium reaching the plastid will activate specific lipoxygenases there as the first committed step of the oxylipin pathway generating jasmonates. 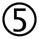 *MYB14* transcription factor. 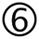 Stilbenes.

In the current study, to understand the functional relevance of these specific promoters and to map the upstream signalling, we employed a promoter–reporter assay (Höll *et al.*, 2013). We show that both *sylvestris* promoters (but not the promoter from the weak stilbene producer Augster Weiss) are induced by UV light in cell culture and that this induction requires the activity of a NADPH oxidase. However, only the *MYB14* promoter from Hoe29 was also induced by jasmonic acid and flg22 (again dependent on the activity of a NADPH oxidase) indicating that this allele of *MYB14* was, in addition, a target of the signalling driving basal immunity (PTI). By contrast, the *MYB14* promoter from Ke83, although inducible by UV light, was not induced in the context of PTI. This is consistent with our previous findings (Duan *et al.*, 2015), where *StSy* transcripts were strongly induced by UV light in both genotypes whereas, for infection by *P. viticola*, induction was only observed in Hoe29, but not in Ke83. We will present a signature model of immunity signalling that can explain most, if not all of our observations (Fig. 7).

The earliest known cellular response of basal immunity is the activation of a rapid influx of Ca^2+^ and H^+^ (Nürnberger, 1999). In the case of the PAMP flg22, perception is brought about by the receptor FLS2 (for reviews see Boller and Felix, 2009; Robatzek and Wirthmueller, 2013), for which a grapevine homologue has also been described (Di Gaspero and Cipriani, 2003). An immediate target of activated FLS2 signalling is cyclic-nucleotide gated calcium influx channels (reviewed in Ma and Berkowitz, 2011). Binding of flg22 to the receptor will therefore result within a few minutes in a substantial increase of cytosolic calcium (Fig. 7, 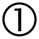).

Calcium, as an important second messenger, plays an important role as an activator of various signal chains (Harper and Harmon, 2005). One of these secondary signalling events is the stimulation of the membrane-bound NADPH oxidase RboH through specific calcium-dependent protein kinases (Kobayashi *et al.*, 2007), leading to an apoplastic oxidative burst generating superoxide anions (Fig. 7, 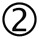). Superoxide anions represent the second central input for plant stress signalling (for a review see Marino *et al.*, 2011), and can also be formed in response to UV light (Hideg *et al.*, 2012). The observation that both *sylvestris* alleles of the *MYB14* promoter show elevated activation by UV that can be blocked by DPI (Fig. 3) suggests that this induction is triggered by RboH. By contrast, only the Hoe29 allele, but not the Ke83 allele, was activated by flg22 (Fig. 6A). This activation was also dependent on the activity of RboH, because it can also be blocked by DPI (Fig. 6B), suggesting that RboH acts downstream of flg22 or complements signalling triggered by flg22. Since activation of RboH by UV light can also activate the Ke83 allele of the *MYB14* promoter, it would be expected, at first sight, that flg22 can also activate this promoter allele because the upstream signalling is provided by the identical recipient (suspension cells of ‘Chardonnay’) in the promoter–reporter system. This means that the differential activation of the two *sylvestris* promoters must be caused by differential activation with other branches of signalling that are independent of RboH. From our model we would predict that these RboH signalling events are activated by calcium influx directly. To test this prediction, we used a calcium ionophore and observed that this significantly activated the Hoe29 allele, whereas the Ke83 and Augster Weiss alleles did not produce significant activation (Fig. 5). When the flg22-triggered influx of calcium is blocked by Gd^3+^ ions, a specific inhibitor of mechanosensitive calcium channels (Ding and Pickard, 1993), this will also block the activation of the Hoe29 allele of the *MYB14* promoter (Fig. 6C). Calcium influx is therefore necessary and sufficient for this activation.

In addition to the activation of RboH through a calcium-dependent protein kinase, cytosolic calcium triggers two additional signalling chains: One of the targets is a MAPK cascade that conveys the signal from the membrane to the nucleus. This MAPK cascade pathway is highly conserved in eukaryotes and is composed of three hierarchical layers whereby MAPK kinase kinases phosphorylate MAPK kinases which, in turn, activate MAP kinases that will then activate different downstream targets. This pathway is central for basal immunity (reviewed in Nürnberger *et al.*, 2004) and also mediates the activation of grapevine stilbene synthase in response to flg22, as concluded from experiments with the specific MAPK inhibitor PD98059 (Chang and Nick, 2012). We therefore tested, whether PD98059 can block the induction of the Hoe29 allele of *MYB14* by flg22. This was what was observed (Fig. 6D), thus showing that the MAPK cascade is necessary.

There is an alternative calcium-dependent branch of defence signalling, though (Fig. 7, 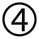): Calcium reaching the plastid will activate specific lipoxygenases there (reviewed in Wasternack and Hause, 2013) as the first committed step of the oxylipin pathway generating jasmonates. In *Arabidopsis*, mutants affected in vacuolar calcium channels (Bonaventure *et al.*, 2007) fail to activate AtLOX2, the lipoxygenase, which is the central trigger for the oxylipin pathway. The molecular mechanism is not clear but might be linked with the binding of lipoxygenase to the membrane due to a conserved calcium binding loop interspersed between two β-sheets (Tatulian *et al.*, 1998). This will alter the specificity of lipoxygenase – whereas the free enzyme converts linoleic acid to conjugated dienes, however, upon binding to the membrane, it preferentially forms a conjugated ketodiene. In consequence, within a few minutes, *cis*-(+)-12-oxophytodienoic acid (OPDA) is exported from the plastid and converted to jasmonic acid (JA) and its potent conjugate JA-Ile.

We therefore tested, whether exogenous jasmonic acid could activate *MYB14* in the absence of flg22. This is, in fact, what we can observe (Fig. 4A), whereby this activation only works with the Hoe29 allele, whereas the alleles from Ke83, and Augster Weiss are not responsive to jasmonic acid. To test, whether induction of (endogenous) jasmonic acid is necessary for this activation, we treated the cells with phenidone, an inhibitor of jasmonate synthesis targeted to lipoxygenases (Ismail *et al.*, 2012), and we found that phenidone can block the flg22-induced activation of *MYB14*. Thus, jasmonate is necessary and sufficient to convey the activation of flg22 to the Hoe29 allele.

This points to a scenario where flg22 activates the MAPK cascade, as well as jasmonate signalling, that converge on a target on the *sylvestris MYB14* promoter that is present in Hoe29, but not in Ke83. Nevertheless, RboH seems to be necessary as well and this effect of RboH seems to be different from that in the UV-activation of *MYB14* (which was similar in both *sylvestris* alleles of this promoter). This apparent discrepancy can be resolved when RboH dependent signalling converges with jasmonate synthesis. This point of convergence might again be the lipoxygenase that is not only activated by calcium, but requires hydrogen peroxide (Fig. 7, 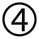). Hydrogen peroxide is generated in the peroxisomes from superoxide and is then further converted to water by catalase (Fig. 7, 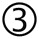). It has been known for a long time that the activity of catalase can be inhibited by the important stress factor salicylic acid, leading to elevated levels of hydrogen peroxide (Durner and Klessig, 1996).

Our model would therefore predict that salicylic acid, by blocking the reduction of hydrogen peroxide, should promote the activation of lipoxygenase and, therefore, the activation of the Hoe29 allele of *MYB14* by flg22 should also depend on RboH. This prediction was tested experimentally and it was found that DPI can block the activation (Fig. 6B), consistent with the prediction. We have further found that salicylic acid can activate the Hoe29 allele of *MYB14* (Fig. 4B). However, the activation was observed for both *sylvestris* alleles pointing to additional targets of salicylic acids (that are different from jasmonate). But, since this activation was also very weak (although significant), the impact of salicylic acid alone (i.e. without synergy with flg22) seems to be fairly marginal.

### Open questions and outlook

The current work proposes a mechanism to explain the observed phenotype (Duan *et al.*, 2015) of a specific *sylvestris* genotype, Hoe29, and draws a link between specific regions in the promoter of the transcription factor *MYB14*, elevated inducibility of this promoter by the signalling activated during basal immunity, and the observed strong accumulation of resveratrol and viniferins correlated with the improved tolerance of these genotypes against downy mildew. Although we can reproduce in the promoter–reporter system the response patterns observed in the plant, for instance the differential activation of stilbene synthase transcription in the Hoe29 versus the Ke83 genotypes, the general activation of the promoter is lower than the observed accumulation of transcripts *in planta*. This indicates that differentiated grapevine cells harbour enhancing factors that are not present in non-differentiated suspension cells. A similar phenomenon with similar ratios is observed for stilbene synthase when the induction of transcripts *in planta* is compared with the inductions observed in the promoter–reporter system (Höll *et al.*, 2013). Whether these factors are of epigenetic nature or are simply additional signalling factors remains to be elucidated. In addition, the role of the *MYB15* factor should be addressed as well as the *MYB14*-independent direct signalling to the stilbene synthase promoter. These aspects are currently being analysed and are expected to complement and refine the proposed working model. As proof of the concept for the approach, the Hoe29 allele of *MYB14* will also be transformed into a vinifera host with poor stilbene performance to see whether up-regulation of *StSy* transcription is sufficient to produce high levels of bioactive stilbenes. The target of this research is to define targets for molecular breeding of grapevine varieties with elevated basal immunity due to enhanced *MYB14* inducibility.

## Supplementary data

Supplementary data can be found at *JXB* online.

Figure S1. The alignment and the significant differential *cis*-elements for the *MYB14* promoters of Hoe29 and Ke83, compared with Augster Weiss and the reference genome (from the *vinifera* cultivar Pinot Noir).

Table S1. Sequence of oligonucleotide primers for GATEWAY^®^ cloning of the *MYB14* constructs for the promoter-reporter assay.

Supplementary Data
