# Supplementary material for: An ancestral allele of grapevine transcription factor MYB14 promotes plant defence
Source: J Exp Bot. 2016 Feb 2;67(6):1795–804. doi: 10.1093/jxb/erv569 (PMC4783363; doi:10.1093/jxb/erv569)
Supplement: Supplementary Data [file supp_67_6_1795__index.html]

An ancestral allele of grapevine transcription factor MYB14 promotes plant defence — An ancestral allele of grapevine transcription factor MYB14 promotes plant defence — Supplementary Data 

# An ancestral allele of grapevine transcription factor *MYB14* promotes plant defence

## Supplementary Data

Data files

- supplementary\_figure\_S1\_table\_S1.pdf - Supplementary Data
