# Supplementary material for: An ancestral allele of grapevine transcription factor MYB14 promotes plant defence
Source: J Exp Bot. 2016 Feb 2;67(6):1795–804. doi: 10.1093/jxb/erv569 (PMC4783363; doi:10.1093/jxb/erv569)
Supplement: Supplementary Data [file supp_erv569_supplementary_figure_S1_table_S1.pdf]

An ancestral allele of grapevine transcription factor *MYB14* promotes plant defence

Dong Duan, Sabine Fischer, Patrick Merz, Jochen Bogs, Michael Riemann, and Peter Nick

Figure S1. The alignment and the significant differential *cis*-elements for the *MYB14* promoters of Hoe29 and Ke83, compared to Augster Weiss and the reference genome (from the *vinifera* cultivar Pinot Noir).

```
Hoe29         CTACTGACGTGCACTAGCCTCTTTCTTTGACCCCTTCACACCATCGATGCTAAATTCCAA 60
Ke83          CTACTGACGTGCACTAGCCTCTTTCTTTGACCCCTTCACACCATCGATGCTAAATTCCAA 60
Augster Weiss CTACTGACGTGCACTAGCCTCTTTCTTTGACCCCTTCACACCATCGATGCTAAATTCCAA 60
Reference     CTACTGACGTGCACTAGCCTCTTTCTTTGACCCCTTCACACCATCGATGCTAAATTCCAA 60
              ************************************************************


Hoe29         GCAGCCCAAATACTTCAACAAATGTGAACTGCACGTGTACACTCTCACACTCGTGTCCAT 120
Ke83          GCAGCCCAAATACTTCAACAAATGTGAACTGCACGTGTACACTCTCACACTCGTGTCCAT 120
Augster Weiss GCAGCCCAAATACTTCAACAAATGTGAACTGCACGTGTACACTCTCACACTCGTGTCCAT 120
Reference     GCAGCCCAAATACTTCAACAAATGTGAACTGCACGTGTACACTCTCACACTCGTGTCCAT 120
              ************************************************************


Hoe29         TTTGTGAATATGGTATTAGGTGTTGTGTTAGGGTCTAATTTTGGGTCGAGTTAAGAGAAC 180
Ke83          TTTGTGAATATGCTATTAGGTTTTGTGTTAGGGTCTAATTTTGGGTCGAGTTAAGAGAAC 180
                              MRE
Augster Weiss TTTGTGAATATGGTATTAGGTGTTGTGTTAGGGTCTAATTTTGGGTCGAGTTAAGAGAAC 180
Reference     TTTGTGAATATGGTATTAGGTGTTGTGTTAGGGTCTAATTTTGGGTCGAGTTAAGAGAAC 180
              ************ ******** **************************************


Hoe29         ACTTATTCATCATACTTTAGCTGGATATGGAAAGTTTTTGAATATGTAATGAAGAAAAGG 240
Ke83          ACTTATTCATCATACTTTAGCTGGATATGGAAAGTTTTTGAATATGTTATGAAGAAAAGG 240
Augster Weiss ACTTATTCATCATACTTTAGCTGGATATGGAAAGTTTTTGAATATGCAATGAAGAAAAGG 240
Reference     ACTTATTCATCATACTTTAGCTGGATATGGAAAGTTTTTGAATATGCAATGAAGAAAAGG 240
              **********************************************  ************

Hoe29         AAAGAAATTTATTTCAAATTTCATCCATTAGTATTTTTATAAATTTATTTTATTTATTAA 300
Ke83          AAAGAAATTTATTTCAAATTTCATCCATTAGTATTTTTATAAATTTATTTTATTTATTAA 300
Augster Weiss AAAGAAATTTATTTCAAATTTCATCCATTAGTATTTTTATAAATTTATTTT--------- 291
Reference     AAAGAAATTTATTTCAAATTTCATCCATTAGTATTTTTATAAATTTATTTT--------- 291
              ***************************************************

Hoe29         ATTTATTTTTTTTAAAGAGATTTAAATGAAAATATTTTAAGTAGAA-----------TCC 349
Ke83          ATTTATTTTTTT-AAAGAGATTTAAATGAAAATATTTTAAGTAGAA-----------TCC 348
Augster Weiss ----------TTTAAAGAGATTTAAATGAAAATATTTTAAGTAGAAGTGTTTAAAAATCC 341
Reference     ----------TTTAAAGAGATTTAAATGAAAATATTTTAAGTAGAAGTGTTTAAAAATCC 341
                        ** *********************************           ***


Hoe29         TCTAATAATTAAATTTTATTCTTTAAAAATTAAAATTTATATATATAAAATATATTTATA 409
Ke83          TCTAATAATTAAATTTTATTTTTTAAAAATTAAAATTTATATATATAAAATATATTTATA 408
Augster Weiss TCTAATAATTAAATTTTATTCTTTAAAAATTAAAATTTATATATATAAAATATATTTATC 401
Reference     TCTAATAATTAAATTTTATTCTTTAAAAATTAAAATTTATATATATAAAATATATTTATC 401
              ******************** **************************************


Hoe29         GGATGTAACCGATAAAATTTGAAATATGTGGCGACTTTATATATTTACATTCAGGTGATG 469
Ke83          GGATGTAACCGATAAAATTTGAAATATGTGGCGACTTTATATATTTACATTCAGGTGATG 468
Augster Weiss GGATGTAACCGGTAAAATTTGAAATATGTGACGATTTTATATATTTACATTAGGGTGGTG 461
Reference     GGATGTAACCGGTAAAATTTGAAATATGTGACGATTTTATATATTTACATTAGGGTGGTG 461
              *********** ****************** *** ****************  **** **


Hoe29         TTTATTTTGCGATTGAATAGAAAAAA-TCAAAATATTTGATTTTTTTTAATTCAATTAAA 528
                                                                CAAT-box
Ke83          TTTATTTTTCGAT----------------------------------------------- 481
Augster Weiss TTTATTTTTTGATTGAATAGAAAAAAATTAAAATATTTGATTTTTCT-AATTTAACTAAA 520
Reference     TTTATTTTTTGATTGAATAGAAAAAA-TCAAAATATTTGATTTTTCT-AATTTAACTAAA 519
              ********  ***


Hoe29         AATAACTCTTTGACATCGTTCAATATAACTAAACTGAACTAATTATTAATAGTTTTAGTT 588
         3-AF1 binding site
Ke83          ------------------------------------------------------------
Augster Weiss AATAACTTGTTCACATCGTTCAATATAACTAAACTGAATTTATTATTAATAGATTCAATT 580
Reference     AATAACTTGTTCACATCGTTCAATATAACTAAACTGAATTTATTATTAATAGATTCAATT 579
```

```
Hoe29        CAATTATGTTGAAAAATATAAACATGTTACTTTTAATTGAATAAAAAAAATCAAATATTT 648
           CAAT-box                           CAAT-box         CAAT-box
Ke83         -AATTATGTTGAAAAATATAAATATGTTACTTTTAACTGAATAAAAAAAGTCAAATATTT 540
             as-2-box                                           CAAT-box
Augster Weiss TAGTTATATTGAAGAATATAAACGGATTACTTTTAACTGAATATAAAAAATTAAGTATTT 640
Reference    TAGTTATATTGAAGAATATAAACGGATTACTTTTAACTGAATATAAAAAATTAAGTATTT 639
              * **** ***** ********    ********** ****** ***** * ** *****

Hoe29        TAATTTTTTATATTCAATACAAAAACAAACACCATTTACATCTCAAGATTATCAATGAAA 708
                                                        circadian
Ke83         TAATTTTTTATATTCAATACAAAAACAAACATCATTTACAACTCAAGATTATCAATGAAA 600
                                                        circadian
Augster Weiss TAATTTTTCATATTCAATAAAAAAACAAACATCACTTAAGATTCA-GATTATCAATGAAA 699
Reference    TAATTTTTCATATTCAATAAAAAAACAAACATCACTTAAGATTCA-TATTATCAATGAAA 698
             ******** ********** *********** ** ***     ***  *************

Hoe29        AACTCAAAATTAAAATATTTCTGCATATATTCTAATGCACCGCCTTAAGATGAGCCGGTT 768
Ke83         AACTCAAAATTAAAATATTTCTGCATATATTCTAAAGCACCGCCTTAAGATGAGCCGGTT 660
Augster Weiss AACTCAAAATTAAAATATTTCTGCATATATTCTAAGGCACCGCCTTAAGATGAGCCTGTT 759
Reference    AACTCAAAATTAAAATATTTCTGCATATATTCTAAGGCACCGCCTTAAGATGAGCCTGTT 758
             *********************************** ******************** ***

Hoe29        ATTCTATTAAAGAGATAATAACGAATTTGAAAAGGCAGAAAAGGAAAATACCAAGAAGGA 828
Ke83         ATTCTATTAAAGAGATAATAACGAATTTGAAAAGGCAGAAAAGGAAAATACCAAGAAGGA 720
Augster Weiss ATTCTATTAAAGAGATAATAACGAATTTGAAAAGGCAGAAAAGGAAAATACCAAGAAGGA 819
Reference    ATTCTATTAAAGAGATAATAACGAATTTGAAAAGGCAGAAAAGGAAAATACCAAGAAGGA 818
             ************************************************************

Hoe29        CTTGGGGATATTGAACGTCACATTCATAGGGATCGCCTTGCAGAAGAAAACAAAAACAAG 888
Ke83         ATTGGGGATATTGAACGTCACATTCATAGGGATCGCCTTGCAGAAGAAAACAAAAACAAG 780
Augster Weiss TTTGGGGATATTGAACGTCACATTCATAGGGATCACCTTGCAGAAGAAAACAAAAACAAG 879
Reference    TTTGGGGATATTGAACGTCACATTCATAGGGATCACCTTGCAGAAGAAAACAAAAACAAG 878
             ********************************* *************************

Hoe29        ATGAAATTCATTCCGGGTAAGTTATTATATAGCAAGTTGGTGCGTTAATTTGCCAAAGTT 948
Ke83         ATGAAATTCATTCCGGGTAAGTTATTATATAGCAAGTTGGTGCGTTAATTTGCCAAAGTT 840
Augster Weiss ATGAAATTCATTCCGGGTAAGTTATTATATAGCAAGTTGGTGCGTTAATTTGCCAAAGTT 939
Reference    ATGAAATTCATTCCGGGTAAGTTATTATATAGCAAGTTGGTGCGTTAATTTGCCAAAGTT 938
             ************************************************************

Hoe29        GGTCACAAGTTTCATTAAAATAATAATAATAATAATAATAATAGGAAAGAAAGAAGGAAA 1008
Ke83         GGTCACAAGTTTTATTAAAATAATAATAATAATAATA------GGAAAGAAAGAAGGAAA 894
Augster Weiss GGTCACAAGTTTCATTAAAATAATAATAATAATAATA------GGAAAGAAAGAAGGAAA 993
Reference    GGTCACAAGTTTCATTAAAATAATAATAATAATAATA------GGAAAGAAAGAAGGAAA 992
             ************ ************************      *****************

Hoe29        AGAAAAATTCTTGAACTCAAATGTAAAATATCTGAACATGCCCCAATTAATGGCCATGCT 1068
Ke83         AGAAAAATTCTTGAACTCAAATGTAAAATATCTGAACACGCCCCAATTAATGGCCATG-T 953
Augster Weiss AGAAAAATTCTTGAACTCAAATGTAAAATATCTGAACATGCCCCAATTAATGGCCATGCT 1053
Reference    AGAAAAATTCTTGAACTCAAATGTAAAATATCTGAACATGCCCCAATTAATGGCCATGCT 1052
             ************************************** ******************* *

Hoe29        AGTTCAAGGAAAAGAAAAACCCCACGTTTTATTTGACCAATAAACAAACACTCGTGTCAT 1128
Ke83         AGTTCAAGGAAAAGAAAAACCCCACGTTTTATTTGACCAATAAACAAACACTCGTGTCAT 1013
Augster Weiss AGTTCAAGGAAAAGAAAAACCCCACGTTTTATTTGACCAATAAACAAACACTCGTGTCAT 1113
Reference    AGTTCAAGGAAAAGAAAAACCCCACGTTTTATTTGACCAATAAACAAACACTCGTGTCAT 1112
             ************************************************************

Hoe29        CCAGTGAGGCGGTTCCTAGCAATTGTGGGCTAAAAAGGATATGCCTTTTTATTTTCTTTT 1188
Ke83         CCAGTGAGGCGGTTCCTAGCAATTGTGGGCTAAAAAGGATATGCCTTTTTATTTTCTTTT 1073
Augster Weiss CCAGTGAGGCGGTTCCTAGCAATTGTGGGCTAAAAAGGATATGCCTTTTTATTTTCTTTT 1173
Reference    CCAGTGAGGCGGTTCCTAGCAATTGTGGGCTAAAAAGGATATGCCTTTTTATTTTCTTTT 1172
             ************************************************************

Hoe29        TTTCTTCTTTTTTTCTAAATTCTATCAACAGTTGTTTGTCCTATCTGCAAATTGCAGCAG 1248
        5'UTR Py-rich stretch                      GATA-motif
Ke83         TTT----------TCTAAATTCTATCAACAGTTGTTTGTACTATCTGCAAATTGCAGCAG 1123
Augster Weiss TTT----------TCTAAATTCTATCAACAGTTGTTTGTACTATCTGCAAATTGCAGCAG 1223
```

```
Reference     TTT----------TCTAAATTCTATCAACAGTTGTTTGTACTATCTGCAAATTGCAGCAG 1222
              ***          ************************* ********************


Hoe29         CTGGACTCCTCATTATAAATACCCGCTCATGGGCTTCAAATCGGTTTGAGCTTGGGACAT 1308
Ke83          CTGGACAGC--ACTATAAATACCCGCTCATGGGCTTCAAATCGGTTTGAGCTTGGGACAT 1181
Augster Weiss CTGGACTCCTCATTATAAATACCCGCTCATGGGCTTCAAATCGGTTTGAGCTTGGGACAT 1283
Reference     CTGGACTCCTCATTATAAATACCCGCTCATGGGCTTCAAATCGGTTTGAGCTTGGGACAT 1282
              ******  *  *  ************************************************


Hoe29         CGACAGAGAACACAG---------------------TCTCAAATCCTTACATAGAGAAGA 1347
                                                               TCA-element
Ke83          CAAGAGAGACCACAGAGATAAACAAGGGTGTCTCAGTCTCAAATCCTTACATAGAAAAGA 1241
Augster Weiss CAAGAGAGAACACAGAGATAAACAAGGGTGTCTCAGTCTCAAATCCTTACGTAGAAAAGA 1343
Reference     CAAGAGAGAACACAGAGATAAACAAGGGTGTCTCAGTCTCAAATCCTTACGTAGAAAAGA 1342
              * * ***** *****                     ************** **** ****

Hoe29         AAAA 1351
Ke83          AAAA 1245
Augster Weiss AAAA 1347
Reference     AAAA 1346
              ****
```

CAAT box: common *cis*-acting element in promoter and enhancer regions.

circadian: *cis*-acting regulatory element involved in circadian control.

Ke83

MRE: *MYB* binding site involved in light responsiveness.

as-2-box: involved in shoot-specific expression and light responsiveness.

Hoe29

3-AF1 binding site: light responsive element.

5'UTR Py-rich stretch: *cis*-acting element conferring high transcription levels.

GATA-motif: part of a light responsive element.

TCA-element: *cis*-acting element involved in salicylic acid responsiveness.

Table S1. Sequence of oligonucleotide primers for GATEWAY® cloning of the *MYB14* constructs for the promoter-reporter assay.

**Name**	**Primer sequence 5'-3'**
Promoter of Hoe29	Sense: 5'- -3'<br>GGGGACAAGTTTGTACAAAAAAGCAGGCTTCCTACTGACGTGCACTAGCCT<br>Antisense: 5'- -3'<br>GGGGACCACTTTGTACAAGAAAGCTGGGTCTTTTTCTTCTCTATGTAAGGATTTGA
Promoter of Ke83	Sense: 5'- -3'<br>GGGGACAAGTTTGTACAAAAAAGCAGGCTTCCTACTGACGTGCACTAGCCT<br>Antisense: 5'- -3'<br>GGGGACCACTTTGTACAAGAAAGCTGGGTCTTTGAGACTGAGACACCCTTG
Promoter of Augster Weiss	Sense: 5'- -3'<br>GGGGACAAGTTTGTACAAAAAAGCAGGCTTCCTACTGACGTGCACTAGCCT<br>Antisense: 5'- -3'<br>GGGGACCACTTTGTACAAGAAAGCTGGGTCTTTGAGACTGAGACACCCTTG
